# Post‐Artesunate Delayed Hemolysis: Anything That Can Go Wrong Will Go Wrong—Murphy's Law

**DOI:** 10.1002/ccr3.70021

**Published:** 2024-12-26

**Authors:** Beliza Chemutai, Muhammed Omar Ali, Jaimin Vinod Varsani, Derrick Bary Abila, Denis Can Odonga, Paska Apiyo, Felix Bongomin

**Affiliations:** ^1^ Department of Internal Medicine Gulu Regional Referral Hospital Gulu Uganda; ^2^ College of Health Sciences Makerere University Kampala Uganda; ^3^ Department of Medical Microbiology & Immunology, Faculty of Medicine Gulu University Gulu Uganda

**Keywords:** artesunate, malaria, *plasmodium*, post‐artesunate delayed hemolysis, Uganda

## Abstract

In patients presenting with post‐malarial anemia following intravenous artesunate treatment, post‐artesunate delayed hemolysis should be considered in the differential diagnosis, even in endemic settings. Close monitoring for signs of delayed hemolysis in patients previously treated with intravenous artesunate for severe malaria, regardless of their malaria exposure history or geographic location is crucial.

## 1 Introduction

Severe malaria is a life‐threatening clinical spectrum of disease associated with multi‐organ dysfunction and is predominantly caused by *Plasmodium falciparum* infection [1, 2]. However, a small proportion of complicated cases are due to other non‐*falciparum plasmodium* species [3, 4, 5]. Globally, over 249 million new cases of malaria are diagnosed, and 608,000 malaria deaths were estimated in 2022 [6]. Most malaria cases occur in low‐ and middle‐income countries. In 2022, the African region accounted for 94% of global malaria cases and 95% of malaria‐related deaths. In 2022, Uganda accounted for 5% of global new cases and 7.8% of malaria deaths [6]. Artesunate is a drug of choice for management of patients with severe malaria. The common side effects of artesunate include hemoglobinuria, jaundice, acute renal failure, paresis, anaphylaxis, and hypersensitivity reaction [7]. Post artesunate delayed hemolysis (PADH) is one of the rare side effects of artesunate. PADH often happens following administration of intravenous artesunate, with the patients having incidences of delayed‐onset hemolytic anemia, usually occurring 7–30 days after the initiation of artemisinin‐based therapy [8, 9, 10, 11]. While most reported cases of PADH occur in non‐immune travelers from malaria‐endemic regions, its occurrence among individuals in endemic areas is less well‐documented. Here in, we report as a rare case of PADH from Uganda. This case is of particular interest because it highlights PADH in a patient with prior malaria exposure in Uganda, a high‐burden setting.

## 2 Case History/Examination

A 45‐year‐old Ugandan female presented to the emergency department with a progressive worsening of symptoms of general body weakness, vomiting, and jaundice over a 2‐day period. A week prior to her current admission, she was managed for severe malaria at a peripheral health facility where she presented with symptoms of fever, joint pain, and general body weakness for a 3‐day period. Her blood slides for *P. falciparum* were positive and Histidine Rich Protein 2 (HRP‐2) rapid diagnostic test was positive too. She was treated with standard dose of intravenous artesunate (3 doses at 0, 12, and 24 h) and was discharged on oral artemether/lumefantrine fixed dose tablets for 3 days as per the Uganda Clinical Guidelines.

In her current presentation, her episodes of vomiting were non‐projectile, non‐bilious, non‐bloody and mainly contained food contents and was not associated with abdominal pain, distention, or early satiety. The yellow discoloration of her mucous membrane was not associated with skin itching or skin discoloration, however, she reported passing tea‐colored urine, with no history of passing frank blood in urine. She denied any history of fevers, dizziness, or headaches. She had no known chronic illness, hematological disorders, food, or drug allergies and had no prior history of recent or past blood transfusion. This was her index episode of afebrile, painless jaundice.

On examination, she appeared sick looking, with severe pallor of the conjunctiva, deep scleral icterus, and some dehydration. There were no visible scratch marks. Her vitals were as follows: blood pressure (118/68 mmHg), pulse rate (112 bpm), respiratory rate (19 breaths per minute), temperature (afebrile) and Glasgow Coma Score of 15/15.

Abdominal examination was normal, with no palpable hepato‐splenomegaly. Respiratory examination was unremarkable with clear chest and normal broncho‐vesicular breath sounds. Cardiovascular examination was with regular pulse rate of 112 bpm, heart sounds 1 and 2 were heard, with no added sounds auscultated.

## 3 Methods (Differential Diagnosis, Investigations and Treatment)

A diagnosis of severe hemolytic anemia with thrombocytopenia as a complication of intravenous artesunate administration was made with key differentials of Evans syndrome and iron deficiency anemia Severe microcytic anemia and thrombocytopenia was noted in full hemogram, however, peripheral film noted normochromic and normocytic red blood cells with morphologically normal platelets. However, both direct and indirect Coombs tests were negative, ruling out Evans Syndrome. Due to resource limitations, LDH and bilirubin lab tests were not conducted. She received two units of packed cells transfusion of matched blood group A+ and was monitored for 72 h. Repeat malaria test was negative. Her work‐up results are summarized in Table 1.

**TABLE 1:** Lab investigations conducted.

Investigation	Parameters	Results	Reference range
Blood smear		No malaria parasites seen	
Peripheral blood film	White blood cells (WBCs)	Normal proliferation	
	Red blood cells (RBCs)	Normocytic, normochromic cells. No target cells or inclusion bodies seen	
	Platelets (PLTs)	Appear normal and adequate	
Complete blood count	Hemoglobin (Hb)	5.8 g	12.0–16.0 g/dL
	WBC Count	4.9	4.5–11.0 × 10^9^/L
	PLT Count	125	150–400 × 10^9^/L
	RBC count	2.3	3.5–5.5 × 10^12^/L
	Mean Corpuscular Volume	54.5	80–100 fL
	Mean corpuscular hemoglobin	24.3	25.4–34.6 pg/cell
	Mean corpuscular hemoglobin concentration	34.6	25.4–34.6 pg/cell
	Post transfusion hemoglobin	7.1 g/dL	
Direct coombs test		Negative	

## 4 Conclusion and Results (Outcome and Follow‐Up)

She showed rapid clinical improvement, with clearance of the jaundice and waning of all admission symptoms. She had no complaints at discharge and was discharged on oral ferrous sulphate 200 mg twice daily for 4 weeks, with vitamin C 500 mg twice for iron deficiency prophylaxis.

## 5 Discussion

PADH is a recognized, though uncommon, complication of intravenous artesunate treatment for severe malaria [12]. This case report presents a 45‐year‐old Ugandan woman with PADH, highlighting the potential for this complication even within malaria‐endemic settings [11, 13]. To the best of our knowledge, this is the first documented case of PADH reported from Uganda. Additionally, the patient's presentation underscores the protean nature of PADH and its potential to mimic other causes of post‐malarial anemia, posing diagnostic challenges in resource‐limited settings. This case emphasizes the importance of recognizing PADH as a potential complication of artesunate treatment, even in endemic regions, and underscores the need for heightened clinical vigilance and further research to better understand its pathogenesis, prevalence, and risk factors in such populations.

PADH can manifest anywhere from at least 7–32 days after intravenous artesunate initiation, characterized by a significant drop in hemoglobin levels, rise in baseline lactate dehydrogenase (LDH) levels, signs of hemolysis, hyperbilirubinemia and the absence of malaria parasites [10, 14, 15]. The patient's presentation aligns with this timeframe, as hemolytic anemia was documented approximately a week after artesunate therapy. Due to resource limitations, LDH and bilirubin lab tests were not conducted.

The differential diagnosis included other conditions that can cause post‐malarial hemolytic anemia. Evans Syndrome, a rare autoimmune disorder characterized by multiple cytopenias, was considered [16]. However, the negative direct antiglobulin test, ruled out this diagnosis. Additionally, the timeframe of the hemolytic episode, aligning with typical delay in PADH, and the lack of findings suggestive of blackwater fever, other infectious triggers further supported PADH as the more likely diagnosis.

While PADH is often reported in non‐immune travelers receiving artesunate [13], this case demonstrates its potential occurrence in patients with prior malaria exposure. The exact prevalence of PADH in endemic regions remains less defined, but systematic reviews suggest a possible lower incidence compared to non‐endemic settings [8, 13, 17]. Literature indicates several potential risk factors, including hyperparasitemia, high cumulative artesunate dose, and elevated bilirubin levels [11, 18].

The underlying mechanism of PADH is still debated. The prevailing pitting theory talks about a splenic role in removing previously infected erythrocytes with shortened lifespans after artesunate‐induced parasite clearance [8, 18]. Further studies are recommended to elucidate on the pathogenesis of PADH, particularly in malaria endemic areas.

Other hypotheses point to ongoing immune activation after the acute malaria phase or artemisinin‐induced metabolic changes within red blood cells. Interestingly, a positive direct antiglobulin test (DAT or Coombs test) had been observed in nearly half of reported PADH cases [17] and is a common feature of Evans Syndrome [16]. While the patient had a negative DAT, this finding might suggest a potential underlying autoimmune component in some PADH cases, warranting further research into the varied mechanisms of PADH.

Patient was successfully managed with blood transfusions to address severe anemia and close monitoring, mirroring the approach described in many PADH cases [12, 18]. The hemolysis was self‐limiting, with the patient showing clinical improvement and resolution of symptoms.

The main limitation of this case report is in the capabilities of laboratories to complete all the relevant investigations to confirm and rule out other differential diagnoses of hemolysis and jaundice. However, this case provides a good insight onto the protean manifestation of malaria and malaria‐treatment associated complications. This is an area of clinical interests requiring large case series and prospective studies.

## 6 Conclusions

There is an increased need of considering PADH as a differential diagnosis of post malarial anemia, even in endemic regions. There is need for further research into PADH, particularly in endemic regions, to better understand its pathogenesis and incidence.

## Author Contributions

**Beliza Chemutai:** conceptualization, data curation, formal analysis, funding acquisition, investigation, methodology, project administration, resources, software, supervision, validation, visualization, writing – original draft, writing – review and editing. **Muhammed Omar Ali:** conceptualization, data curation, writing – original draft, writing – review and editing. **Jaimin Vinod Varsani:** writing – original draft, writing – review and editing. **Derrick Bary Abila:** writing – original draft, writing – review and editing. **Denis Can Odonga:** investigation, writing – original draft, writing – review and editing. **Paska Apiyo:** conceptualization, investigation, writing – original draft, writing – review and editing. **Felix Bongomin:** conceptualization, data curation, formal analysis, funding acquisition, investigation, methodology, project administration, resources, software, supervision, validation, visualization, writing – original draft, writing – review and editing.

## Ethics Statement

A written informed consent was obtained from patient to publish this report in accordance with the journal's patient consent policy.

## Conflicts of Interest

The authors declare no conflicts of interest.

## Data Availability

Data sharing does not apply to this article as no new data was created or analyzed in this study.
